# Assessment of quality of life in children with epilepsy in Oman

**DOI:** 10.1186/s41687-023-00555-1

**Published:** 2023-02-02

**Authors:** Asia Alnaamani, Faraz Ahmad, Muna Al-Saadoon, Syed Gauhar Alam Rizvi, Amna Al-Futaisi

**Affiliations:** 1 Child Health, Department of Woman and Child Health, Ministry of Health, 123 Alkhoud, Muscat, Oman; 2 Department of Child Health, Sultan Qaboos University Hospital, Muscat, Oman; 3 Sultan Qaboos University, P.O. Box 38, 123 Al-Khoudh, Oman; 4 Department of Child Health, College of Medicine and Health Sciences, Sultan Qaboos University, P.O. Box 35, 123 Al-Khoudh, Muscat, Oman; 5 Department of Family Medicine and Public Health, College of Medicine and Health Sciences, Sultan Qaboos University, Muscat, Oman

**Keywords:** Epilepsy, Children, Quality of life, Oman

## Abstract

**Purpose:**

The study aims to describe the quality of life (QoL) in Omani children with epilepsy at Sultan Qaboos University Hospital, Oman.

**Methods:**

One hundred and one Omani children, with an age range from 5 to 18 years, diagnosed with epilepsy were enrolled in the study over 3 months. Descriptive epidemiology was used to characterize QoL in these children. QoL was measured using the PedsQL (4.0) questionnaire, a 23-item child and parent report questionnaire. Analysis of variance (ANOVA) was used to compare mean QoL scores, and agreement between the QoL reports of children and parents was evaluated using Spearman’s rho; while, Multivariate analysis of variance (MANOVA) was performed to determine differences in subscale ratings.

**Results:**

Factors affecting QoL included family status, income level, social security coverage, type of treatment, seizure frequency, age of onset, and seizure-free duration in years. Children between 5 and 7 years and females, in general, were most affected, as reflected by the overall QoL subscale. Consistency between the children's self-reports and parent proxy reports on the PedsQL™ was moderate to low.

**Conclusion:**

Omani children with epilepsy have poor QoL, and their psychosocial function is severely affected. Therefore, QoL should be an important outcome measure in managing children with epilepsy rather than just seizure control.

## Introduction

Epilepsy can seriously impair the quality of life (QoL) of children and youth with epilepsy (CYWE) and their families. Epilepsy affects 325,000 children and adolescents under 15 years and 1% of all youth [1]. This population shows significantly higher emotional, behavioral, social, and academic difficulties rates than youth with other chronic health conditions or youth in general [2]*.* Moreover, dealing with a child’s epilepsy can be stressful for the entire family [3]. Comparative studies show that families of children with epilepsy have more relationship and parenting difficulties resulting in more disrupted environments than families of children with other chronic conditions [4]***.***

The World Health Organization (WHO) [5] defines QoL as an “individual’s perception of their position in life in the context of the culture and value systems in which they live and concerning their goals, expectations, standards, and concerns”. QoL is an important measure in epilepsy, a debilitating condition unique among chronic illnesses owing to its multidimensional impact on psychosocial function [6]. Measuring health-related QoL (HRQoL) is an important outcome indicator when evaluating healthcare interventions and treatments to understand the burden of disease, identify health inequalities, allocate health resources, and conduct epidemiological studies and health surveys [7]. The Pediatric Quality of Life Inventory (PedsQL™) 4.0 Generic Core Scales is a generic HRQoL instrument for children and adolescents developed initially by Varni et al. [8] in US English and Spanish. It measures four domains (physical, emotional, social, and school functioning). It has age- and respondent-specific versions for child self-report for ages 5–18 years and parent proxy reports for ages 2–18 years [8].

It is essential to understand their perspective on the limitations imposed by their illness to fulfill the needs of children [9]. Only a few published studies are available in the Middle East and North African region (MENA) [10, 11]. However, studies investigating QoL of children with epilepsy from the perspective of the children and their parent’s perceptions have not been conducted in the Sultanate of Oman. Therefore, this study aimed to investigate the QoL of children with epilepsy. The study also identifies QoL’s clinical and social determinants in children with epilepsy followed up at a single tertiary hospital in Oman.

## Methods

Based on child and parent reports, a hospital-based descriptive epidemiological approach was used to determine QoL in children living with epilepsy.

The target population of this study consisted of Omani children with an established diagnosis of epilepsy. The sample included all Omani children with epilepsy (age, 5–18 years), who attended the pediatric neurology clinic during the 3 months from December 1, 2012, to February 28, 2013. Children with a recent diagnosis of epilepsy (defined as duration less than 6 months) were excluded. Children who could not reflect on their QoL, namely those with cognitive impairment and those below 5 years, were also excluded. Data were collected from the electronic hospital medical records, which included socio-demographic and clinical variables and a review of the medical records. A validated questionnaire and a hospital medical record review were used to obtain the required information.

The QoL of children with epilepsy was assessed using PedsQL™ version 4.0 for children and parents [12]. Both versions have previously been translated into Arabic, and their validity and reliability were tested [13]. The children’s version consisted of three forms designed for different age groups: young children (5–7 years), children (8–12 years), and teens (13–18 years). The parents’ version was designed to reflect the children's QoL from the parent's perspective.

The Ethics Committee approved the study of Sultan Qaboos University Hospital. Written informed consent was obtained from the guardians of the children before data collection. Children aged between 13 and 18 years were asked to sign a consent form.

Statistical Package for Social Sciences (SPSS) version 20 was used for data entry and analysis. The normality of variables was tested using the Kolmogorov–Smirnov test. A* t*-test and analysis of variance (ANOVA) were used to determine the factors affecting QoL concerning clinical and social parameters. Spearman’s rho was used to determine the agreement between the child, and parent estimated QoL reports. Multivariate analysis of variance (MANOVA) was performed to determine differences in subscale ratings based on age group, sex, seizure frequency, and age of onset of seizures. Results were considered significant at *p* ≤ 0.05.

## Results

The socio-demographic and clinical variables associated with total QoL scores are shown in Tables 1 and 2 respectively. The results showed that 24.8% of the children (25 /101) were in the age group of 5–7 years, 53.5% (54/101) were in the age group of 8–12 years, and 21.8% (22/101) were in the age group of 13–18 years. Ninety children (89.1%) had primary epilepsy; secondary types were observed in only 11 (10.9%) children. The mean age of the cohort was 10 years, and 54.5% of the patients were male. Focal-onset seizures with retained awareness were the most common type of seizures in the study group (54.55%), followed by focal-onset seizures with impaired awareness (28.71%) and generalized-onset seizures in 27 patients (26.7%). Most children (58.4%) were well-controlled on monotherapy, while polytherapy was reported in 29.7% of the cases. Twelve children (11.9%) were untreated.

**Table 1:** Socio-demographic with total parent reported QoL

Characteristics	Cohort	n (%)	Mean QoL (parent reported)	SD	*p*
Family status*	Intact	94 (93.1%)	79.76	15.25	0.001
	Divorced/Separated	4 (4%)	51.36	30.79	
Family income	< 500	39 (38.6%)	72.27	21.54	0.011
	500–1000	35 (34.7%)	82.20	10.55	
	< 1000	27 (26.7%)	81.84	13.58	
CBSS (covered by social security)	No	83 (82.2%)	81.43	14.73	0.001
	Yes	18 (17.8%)	66.67	20.61	
Age group (years)	5–7	25 (24.8%)	73.09	14.19	0.128
	8–12	54 (53.5%)	79.65	18.29	
	13–18	22 (21.8%)	82.87	17.67	

*Missing data was excluded from analysis

**Table 2:** Clinical variables with total parent reported QOL

Characteristics	Cohort	n (%)	Mean QOL (parent reported)	SD	*p*
Seizure frequency	< Month	21 (20.8%)	65.43	21.05	< 0.0005
	Monthly	31 (30.7%)	78.44	13.64	
	Yearly	17 (16.8%)	82.54	18.01	
	More than1 per Year	32 (31.7%)	85.33	10.81	
Treatment	No-treatment	12 (11.9%)	83.97	9.24	0.011
	Mono-therapy	59 (58.4%)	81.39	15.69	
	Poly-therapy	30 (29.7%)	70.92	19.96	
Hospitalization	Yes	61 (60.4%)	75.61	17.79	0.024
	No	40 (39.6%)	83.37	14.33	
Emergency visit	Yes	75 (74.3%)	76.34	17.21	0.017
	No	26 (25.7%)	85.49	19.39	
Seizure free (in years)*	Less than 1	53 (52.5%)	73.08	19.39	0.009
	1–2	11 (10.9%)	86.96	5.34	
	≥ 3	6 (5.9%)	89.43	12.68	
Age of onset of seizures (years)	≤ 3	29 (28.7%)	70.53	20.34	0.003
	4–7	45 (44.6%)	79.51	16.01	
	> 8	27 (26.7%)	85.67	10.04	

*Missing data was excluded from analysis

**Fig. 1:**
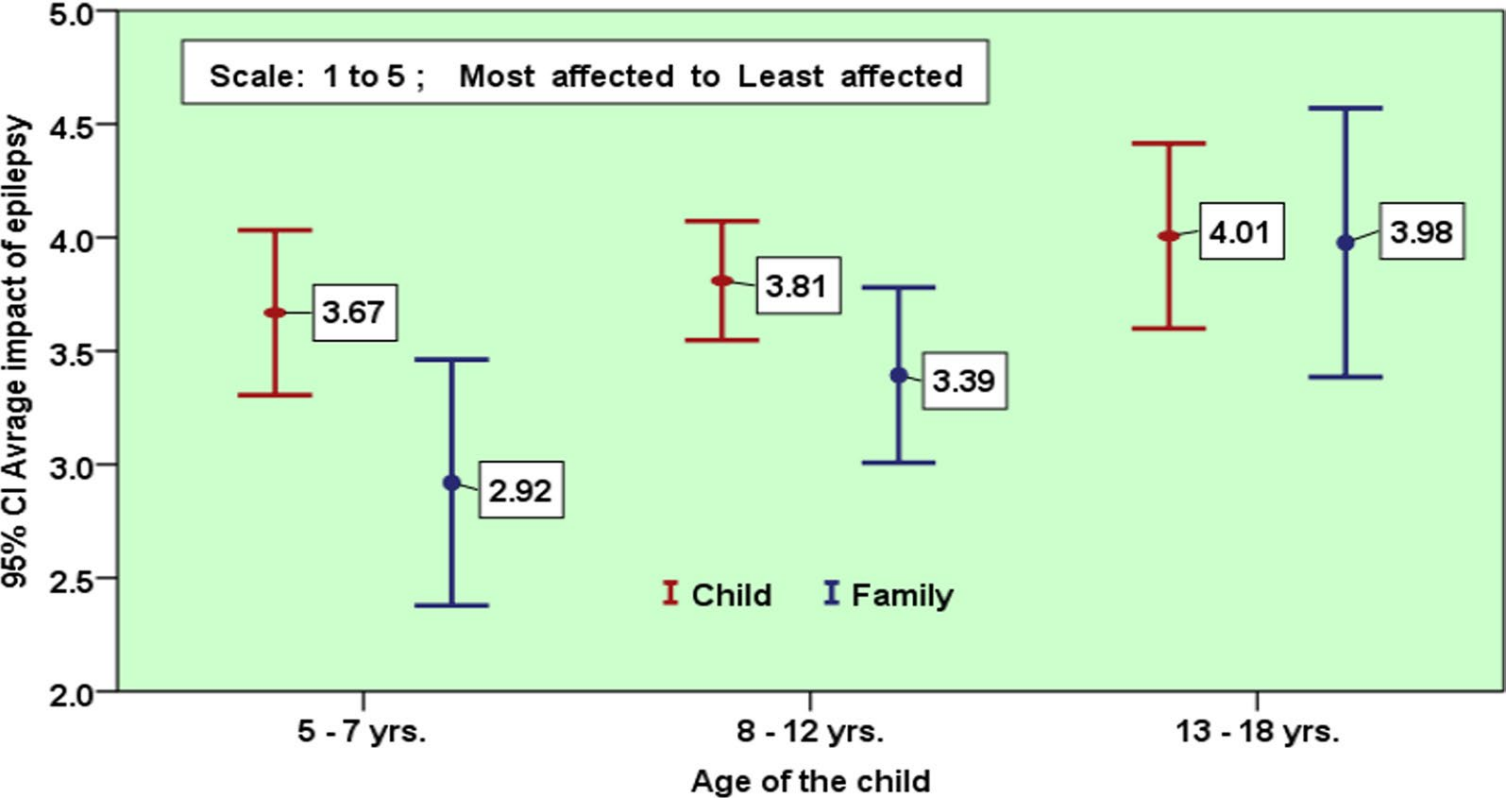
Child and family reported QoL in the different age groups

**Fig. 2:**
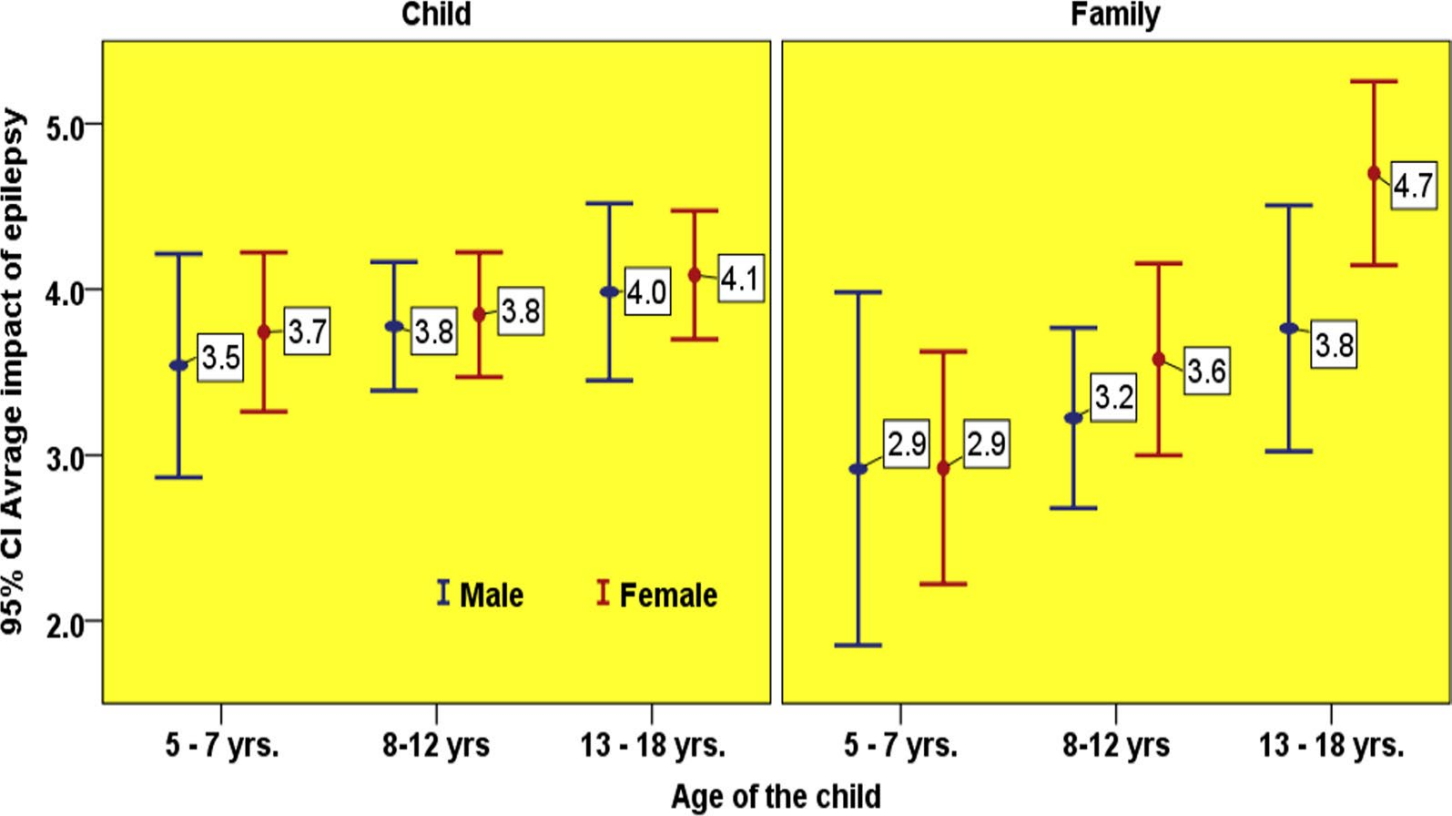
Child and family reported QoL based on gender

**Fig. 3:**
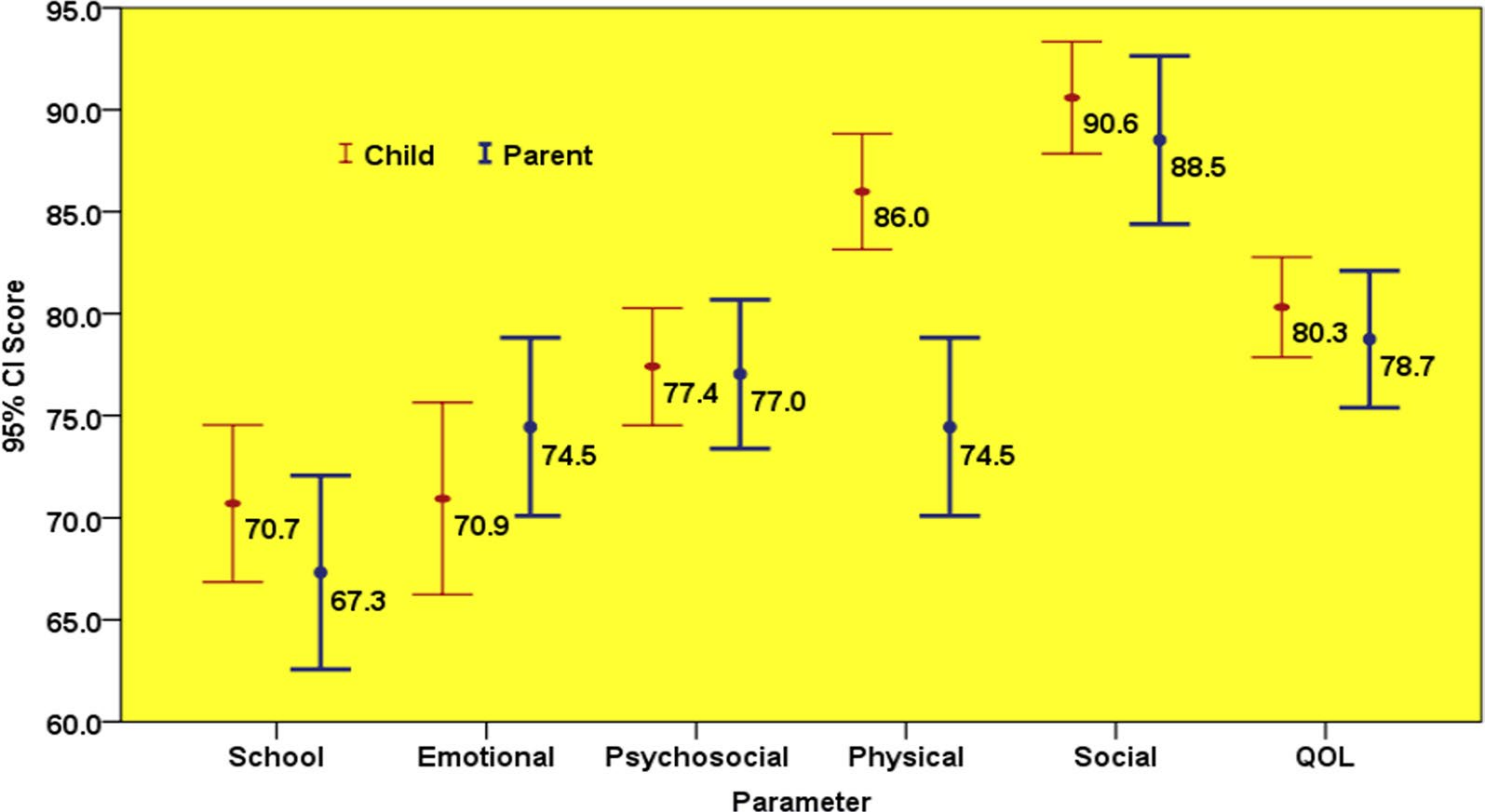
Comparison of child and parent reported QoL in different domains

In general, the effect of epilepsy on the QoL of children was inversely related to the child’s age. The QoL in the youngest age group (5–7 years) was the worst affected by epilepsy, and the QoL in the 13–18 years-old group was the least affected. This trend was consistent with the parent’s reports.. Parent reported QoL score was considerably lower in the age group 5–7 years (Fig. 1). There was no significant difference between the genders, except for noticeable higher parent reported QoL scores for female adolescents (Fig. 2). A comparison of child and parent QoL scores in different domains showed a general trend of lower parent reported QoL scores in contrast with the child QoL scores (Fig. 3).

Among all domains, school functioning showed the lowest QoL score, and social functioning showed the highest score from the perspective of both children and parents. The physical function score observed a highly significant difference between parent and child reported QoL. The overall QoL scores of the children and parental reports were significantly and positively related. On comparing the different domains separately, school functioning demonstrated the strongest relationship with QoL, followed by physical, psychosocial, and social functions. The corelation between children's and parentalreported scores for emotional functioning was weak and insignificantly related (*p* = 0.088).

The parent reported QoL of their children was higher among the parents of older children. Among all four domains, the lowest QoL score reported by parents (55.4) was observed for school functioning in the 5–7-years-old age group. The parent reported QoL was lower among parents of girls, except for school functions, which showed a small difference in favor of girls. The maximum gender-related difference in the QoL scores reported by parents was observed for emotional function, with statistically significant difference (male average = 79.6, female average = 68.5, *p* = 0.012).

Polytherapy with antiseizure drugs was significantly associated with lower total QoL scores (*p* = 0.011) (Table 2).

The parent reported QoL score of untreated children (mean = 83.97) and children on monotherapy regimen (mean = 81.39) were higher than children on the polytherapy regimen (mean = 70.92). Seizure-free duration and overall QoL scores were positively correlated (*p* = 0.009). Children with seizure freedom for ≥ 3 years had better QoL scores (mean = 89.43) than those with seizure-free periods of 1–2 and “< 1 year” (*p* = 0.009).

**Fig. 4:**
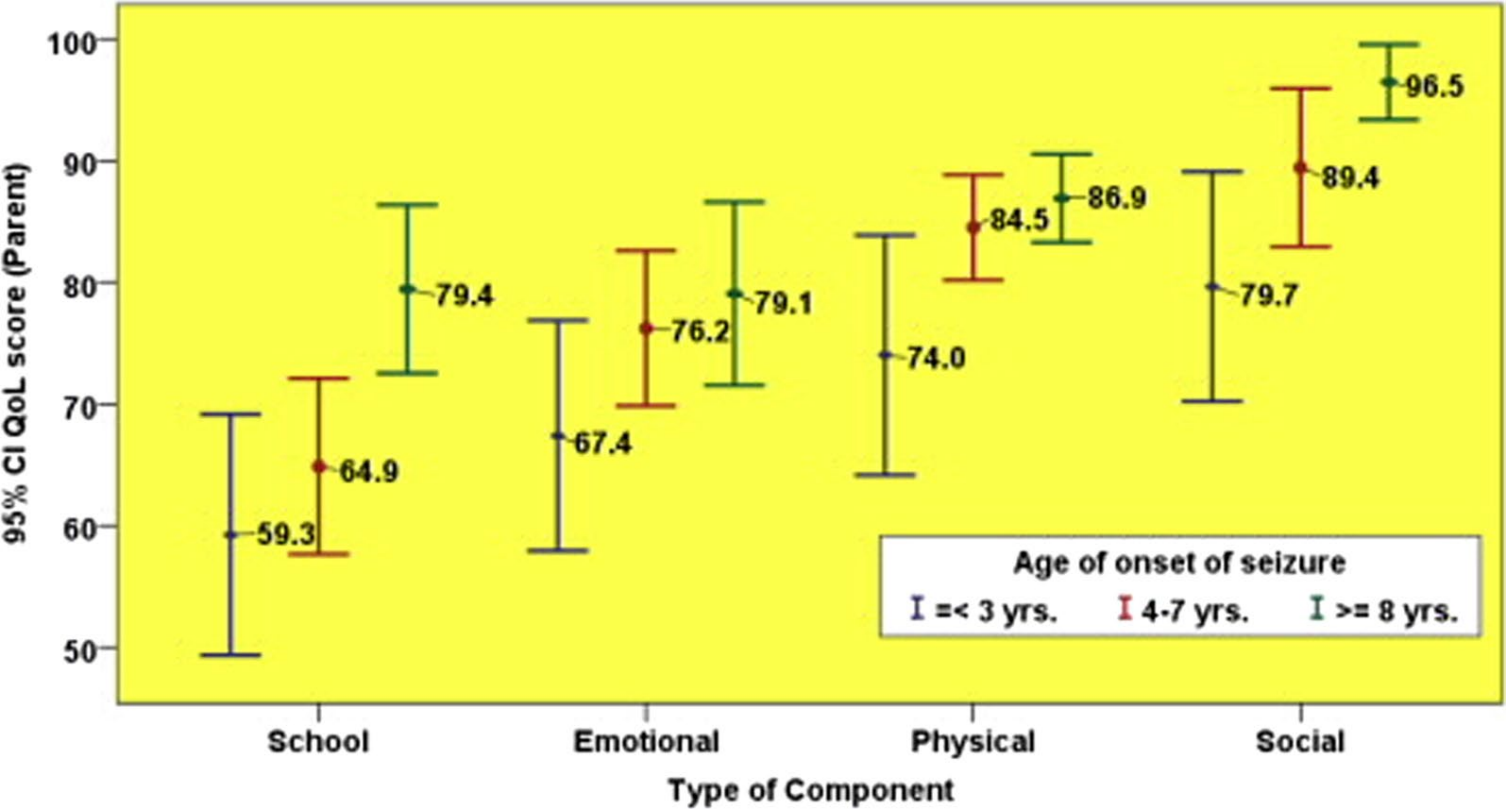
Parent reported QOL in different components based on the age of onset of seizures

Seizure frequency was categorized as “< a month,” “monthly,” “yearly," and “> a year.” Higher seizure frequency had an adverse influence on the QoL reported by parents. The study sample was further categorized into three groups based on the age at epilepsy onset (in years): “≤ 3 years,” “4–7 years," and “≥ 8 years”. The numbers of patients in the three groups were 29 (28.7%), 45 (44.6%), and 27 (26.7%), respectively. Parent reported QoL score was affected by the age of onset in all four domains, suggesting that the earlier the age of onset of epilepsy, the greater the adverse effect on QoL (Fig. 4).

## Discussion

The present study revealed that the average impact score ranged from 3.67 to 4.01 on a scale of 1–5, where 1 reflects the maximal impact, and 5 indicates the least impact. This could be attributed to the sociocultural factors in Omani society, similar to that previously noted by Sherman et al. [14]. The study by Sherman et al. [14] suggested that positive attitudes may be integral to sociocultural factors since the chronic course of illness in traditional communities is accommodated with sympathetic acceptance and assisted culturally.

Based on the scale average of (1–5), the present study found that Omani children aged between 5 and 7-years were the most affected by epilepsy. The influence of epilepsy on the child QoL was higher in parent reported scores compared to those of the child’s him/herself. A possible explanation for this is that parents are likely to be concerned about their children's independence, future education, and marriage. Apart from caring for those with chronic illnesses, epilepsy restricts parents' social activities since they must dedicate more attention and time to their children than usual, even to other siblings.

The parent reported QoL was worse when the affected child was younger. This trend may be accounted for by parental coping strategies, which may be modified by providing appropriate support. Parents with female and older children with epilepsy reported better scores likely because older children are relatively more independent and can care for themselves. Family status was also an important factor that significantly affected QoL in Omani children with epilepsy. Previous studies by Shakir et al. [15] and Chung et al. [16] support this finding. Children who belonged to intact families had better QoL scores than those from divorced or separated families (QoL scores of 79 vs. 51, *p* = 0.001). This is likely due to the loss of income and limited family support, leading to delayed or inadequate access to healthcare and social support services. Shakir et al. [15] reported that low-income levels significantly affect overall QoL.

In the present study, QoL was the lowest in children whose seizures began before 3 years (*p* = 0.003). Early childhood is important for brain development and the maturation of interaction and socialization skills, behavior, and decision-making [17]. Poorly controlled seizures are likely to impair the normal developmental process. Frequent seizures were the most significant clinical factor affecting QoL (*p* < 0.0005), and this finding was consistent with several previous studies [18–21].

Children with polytherapy have lower QoL scores due to the contributory adverse effects of multiple anti-seizure medications in achieving seizure control [22]. Similarly, poor control of seizures is also associated with lower QoL scores, similar to one of the previous studies [23]. Children with seizures in the preceding year had lower QoL scores than those who had been seizure-free for ≥ 1 year [24]. A significant difference (*p* = 0.007) between the QoL scores of primary and secondary epilepsies was also observed. A similar finding was reported by Aggarwal et al. [25], using data obtained with the quality of life in epilepsy questionnaire (QoLIE) and hospitalization history. Children who were frequently hospitalized (poorly controlled epilepsy) had lower QoL scores than those who were not hospitalized for epilepsy (p = 0.024).

Seizure frequency was one of the most significant factors related to QoL (*p* < 0.0005). All other domains showed a clear trend concerning seizure frequency. Children with the most frequent seizures (“< a month”) had poorer QoL scores in all subscale domains [26]. The most affected subscale was school functioning, which was consistent with the findings reported by Serdari et al. [27] and McLaughlin et al. [28]. This may be due to frequent absences from school due to seizures, adversely affecting performance. Age at the onset of seizures has the most significant impact on QoL [29]. Parent reported QoL was significantly lower in all subscale domains among children with seizure onset as early as ≤ 3 years of age Overall, the parent reported QoL score (mean = 78.3) was lower than the child’s QoL score (mean = 80.3). Similarly, Verhey et al. [30], using the epilepsy-specific measure, also reported that parents rated children’s HRQoL as low compared to the self-report provided by the children. Felder-Puig et al. [31] used the German version of the generic PedsQL and observed that parental reports of HRQoL were lower than child reports of HRQoL (74.5 and 86.0, respectively). Emotional and school functioning are usually major concerns of both parents and children. Parents of one-third of all examined patients with epilepsy reported school problems due to disturbed memory and poor attention. Austin et al. [32] reported that QoL in children with epilepsy was more compromised than in children with asthma, particularly in the psychological and school domains. Similar results were also reported by Talarska et al. [33].

Correlations between child self-report and parental report ranged from moderate (*r* = 0.558) for school functioning to very weak (*r* = 0.163) for the emotional functioning subscale. Cremeens et al. [34] investigated the agreement between child and parent proxy reports using the PedsQL (4.0) and found a weak agreement between these scores. The child may feel well-adjusted in contrast to parents who are worried about their child’s social, cognitive, and emotional challenges while comparing their children’s performance with that of the rest of the class. In our study the correlation for each subscale was highly significant (*p* < 0.0005), except for emotional function (*p* = 0.088).

The present study found that parents reported the lowest QoL scores in the youngest age group (5–7 years) in all subscale domains: school, emotion, physical, and social, consistent with similar observations reported in other studies [35, 36]. A possible explanation may be that parents are more stressed and their load of care increases when they have younger children who are unable to articulate their symptoms. The overall trend showed that the QoL score increased as the child got older.

**Fig. 5:**
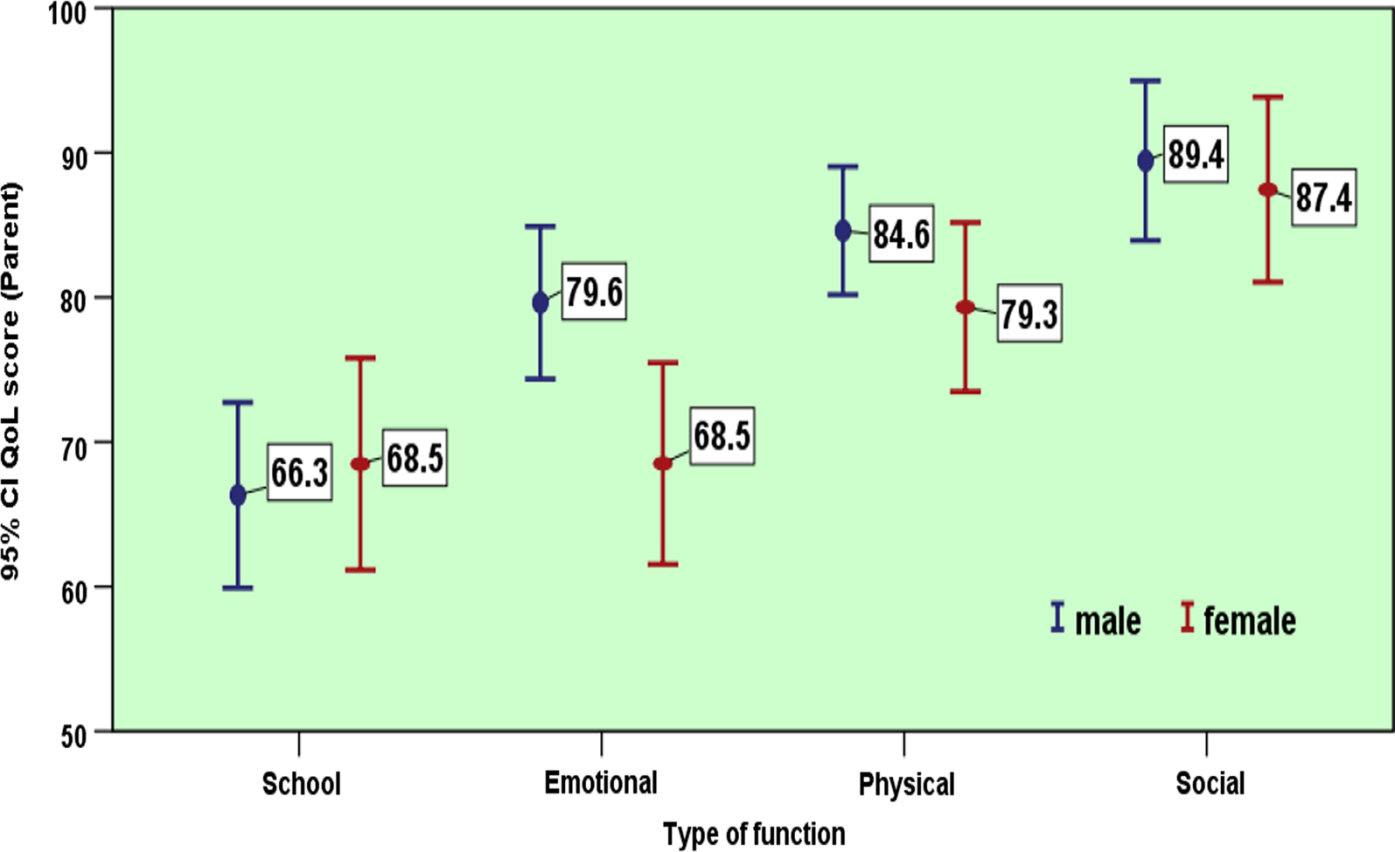
Parent reported QoL in different components based on gender

Overall, parent reported QoL subscale scores were lower in females than males, except in school functioning (Fig. 5), with a marginal difference between the averages (males, 66.3 and females, 68.5). This was inconsistent with the results of Weis et al. [37] who showed that girls in the fifth grade of school outperformed boys in German. Possible reasons for the lower QoL scores for girls in the present study may be that parents are more concerned about their marriage and future responsibilities after marriage.

## Conclusion

In conclusion, Omani children with epilepsy have compromised QoL due to socio-demographic factors (family status, family income level, and coverage by social security) as well as clinical factors (seizure frequency, age of onset, seizure-free years, type of epilepsy, type of treatment, hospitalization, and comorbid illnesses). Poorly controlled seizures and early-onset seizures (< 3 years) were associated with poor functioning in all domains of the PedsQL (4.0) score, contributing to a more significant decline in school and emotional functioning. Emotional and school functioning were of the most critical concern to children and parents.

The availability of psychological care and social support for families is expected to help improve the QoL of the family unit. However, controlled trials of psychological interventions in this group are lacking. Future controlled trials of such interventions may provide more robust evidence of their impact on the QoL of children with epilepsy and their families in Oman.

## Data Availability

Not applicable.

## Abbreviations

ANOVA: Analysis of variance
CYWE: Children and youth with epilepsy
HRQoL: Health-related quality of life
MANOVA: Multivariate analysis of variance
PedsQL: The Paediatric Quality of Life Inventory
QoL: Quality of life
QoLIE: Quality of life in epilepsy questionnaire
CBSS: Covered By Social Security
